# Progesterone Receptor-Mediated Regulation of N-Acetylneuraminate Pyruvate Lyase (NPL) in Mouse Uterine Luminal Epithelium and Nonessential Role of NPL in Uterine Function

**DOI:** 10.1371/journal.pone.0065607

**Published:** 2013-05-31

**Authors:** Shuo Xiao, Rong Li, Honglu Diao, Fei Zhao, Xiaoqin Ye

**Affiliations:** 1 Department of Physiology and Pharmacology, College of Veterinary Medicine, University of Georgia, Athens, Georgia, United States of America; 2 Interdisciplinary Toxicology Program, University of Georgia, Athens, Georgia, United States of America; 3 Reproductive Medical Center, Renmin Hospital, Hubei University of Medicine, Shiyan, Hubei, China; Medical Faculty, Otto-von-Guericke University Magdeburg, Medical Faculty, Germany

## Abstract

N-acetylneuraminate pyruvate lyase (NPL) catalyzes N-acetylneuraminic acid, the predominant sialic acid. Microarray analysis of the periimplantation mouse uterine luminal epithelium (LE) revealed *Npl* being the most downregulated (35×) gene in the LE upon embryo implantation. In natural pregnant mouse uterus, *Npl* expression increased 56× from gestation day 0.5 (D0.5) to D2.5. In ovariectomized mouse uterus, *Npl* was significantly upregulated by progesterone (P4) but downregulated by 17β-estradiol (E2). Progesterone receptor (PR) antagonist RU486 blocked the upregulation of *Npl* in both preimplantation uterus and P4-treated ovariectomized uterus. *Npl* was specifically localized in the preimplantation D2.5 and D3.5 uterine LE. Since LE is essential for establishing uterine receptivity, it was hypothesized that NPL might play a critical role in uterine function, especially during embryo implantation. This hypothesis was tested in the *Npl* ^(−/−)^ mice. No significant differences were observed in the numbers of implantation sites on D4.5, gestation periods, litter sizes, and postnatal offspring growth between wild type (WT) and *Npl* ^(−/−)^ females from mating with WT males. *Npl* ^(−/−)^x*Npl* ^(−/−)^ crosses produced comparable little sizes as that from WTxWT crosses. Comparable mRNA expression levels of several genes involved in sialic acid metabolism were observed in D3.5 uterus and uterine LE between WT and *Npl* ^(−/−)^, indicating no compensatory upregulation in the D3.5 *Npl* ^(−/−)^ uterus and LE. This study demonstrates PR-mediated dynamic expression of *Npl* in the periimplantation uterus and dispensable role of *Npl* in uterine function and embryo development.

## Introduction

N-acetylneuraminate pyruvate lyase (NPL), also named sialic acid aldolase or N-acetylneuraminate lyase, was originally purified and characterized in human related pathogenic as well as non-pathogenic bacteria that utilize the carbon sources in the mucus-rich surfaces of the human body, such as *Clostridium perfringens* and *Escherichia coli* [1]–[4]. The human NPL consists of 320 amino acids (33 kDa) and has a crystal structure of tetramer [5], [6]. Mammalian NPL proteins have 86 highly conserved amino acids, which are slightly different from the bacterial counterpart [6]. A splice variant of human *NPL* is highly expressed in human liver, kidney, ovary, and peripheral blood leukocyte [7].

NPL catalyzes the breaking of carbon-carbon bonds of N-acetylneuraminic acid, the predominant sialic acid, into N-acetylmannosamine and pyruvate, thus regulating the cellular concentrations of sialic acid and preventing the recycling of sialic acid for further sialiation with glycoconjuates in the Golgi compartment [8], [9]. In both bacteria and mammalians, sialic acids such as N-acetylneuraminic acid (Neu5Ac) and N-glycolylneur aminic acid (Neu5Gc) are involved in the sialylation and provide the diversity of sialylated oligosaccharides [10]–[12]. Sialic acids have been associated with intercellular adhesion, protein recognition, and immune-related mechanisms [13], [14].

Limited reports suggest that NPL may have functions in female reproduction but the potential role of NPL in female reproduction has not been previously investigated. For example, serum sialic acid level increases with the progression of pregnancy compared with that in non-pregnant women [15]–[17]; uterine sialic acid concentration decreases upon ovariectomy in Indian langur monkeys, but increases upon ovarian hormones E2 or E2+P4 treatments [18]. NPL came to our attention from our microarray analysis of mouse periimplantation uterine luminal epithelium (LE) (GEO number: GSE44451). *Npl* was the most downregulated (35×) gene in the postimplantation gestation day 4.5 (D4.5) LE compared with that in the preimplantation D3.5 LE (Xiao et al, submitted). Further analysis indicated peak expression of *Npl* in the preimplantation D2.5 and D3.5 uterine LE. Since LE is critical for the receptive sensitivity of the uterus [19], [20], we hypothesized that NPL might be involved in uterine function, especially uterine preparation for embryo implantation. This hypothesis was tested in *Npl* ^(−/−)^ mice.

## Materials and Methods

### Animals and genotyping

*Npl* ^(−/−)^ mice were generated from the mouse strain B6/129S5-*Npl^Gt(IRESBetageo)332Lex^*/Mmucd (identification number 011743-UCD) and purchased from the Mutant Mouse Regional Resource Center (MMRRC) at UC Davis, a NCRR-NIH funded strain repository. *Npl* ^(−/−)^ mice were genotyped using tail genomic DNA and three primers in PCR reactions: Primer 0920-5′: GGCATATATGTGCAGGCAGAATGC, Primer LTR-rev: ATAAACCCTCTTGCAGTTGCATC, and Primer 0920-3′: TCTAGAAATGAGTCTGAACCGGAC. The genotyping PCR cycles were: 10 cycles of 94°C for 15s, 65°C for 30 s (decreased 1°C/cycle), 72°C for 40 s; and 30 cycles of 94°C for 15 s, 55°C for 30 s and 72°C for 40 s. The expected PCR product sizes for wild type (WT) (Primer 0920-5′ and Primer 0920-3′) and *Npl* ^(−/−)^ (Primer 0920-5′ and Primer LTR-rev) were 115 bp and 163 bp, respectively. All mice were housed in polypropylene cages with free access to regular food and water from water sip tubes in a reverse osmosis system. The animal facility is on a 12-hour light/dark cycle (7:00 AM to 7:00 PM) at 23±1°C with 30–50% relative humidity. All methods used in this study were approved by the University of Georgia Institutional Animal Care and Use Committee (IACUC) and conform to the National Institutes of Health guidelines and public law. All the animal studies were summarized in Table 1.

**Table 1:** Summary of the mouse studies.

Experiment		Genotype	Age or gestation day (D)	Treatment
Uterine *Npl* mRNA expression in early pregnancy		Wild type	D0.5, D1.5, D2.5, D3.5, D4.5, D5.5, D7.5	None
PR-mediated uterine *Npl* mRNA upregulation in early pregnancy		Wild type	D2.5	Vehicle
				ICI 182780
				RU486
Hormonal regulation of *Npl* mRNA in ovariectomized uterus	Set A	Wild type	6 weeks old	Vehicle
				P4
				E2
				P4+E2
Hormonal regulation of Npl mRNA in ovariectomized uterus	Set B	Wild type	6 weeks old	Vehicle
				P4
				P4+RU486
				RU486
NPL on embryo implantation		Wild type *Npl* ^(−/−)^	D4.5, D5.5, D7.5	None
NPL on gestation period and litter size		Wild type *Npl* ^(+/−)^ *Npl* ^(−/−)^	2∼4 months old	None
NPL on gene expression in uterus and uterine luminal epithelium		Wild type *Npl* ^(−/−)^	D3.5	None

### Mating and uterine tissue collection

Young virgin females were mated naturally with WT stud males and checked for a vaginal plug the next morning. The day a vaginal plug identified was designated as gestation day 0.5 (D0.5, mating night as D0). Uterine tissues were collected from euthanized females between 11:00 h and 12:00 h on D0.5, D1.5, D2.5, D3.5, D4.5, D5.5, and D7.5, respectively. Uterine horns from D0.5 to D2.5 females were quickly removed and snap-frozen on dry ice. Oviducts from these mice were flushed with 1×PBS for the presence of eggs or fertilized embryos to determine the pregnancy status. About 1/3 of a uterine horn from each euthanized D3.5 female was frozen on dry ice for tissue sectioning. The remaining D3.5 uterine horns were flushed with 1×PBS (to determine the status of pregnancy and to remove the influence of embryos on uterine gene expression) and frozen on dry ice for RNA isolation. On D4.5, D5.5, or D7.5, mice were anesthetized with isoflurane by inhalation and intravenously (i.v.) injected with Evans blue dye to visualize the implantation sites as previously described [21]. At least three pregnant mice were included in each group.

### Hormonal treatment

Progesterone (P4), 17β-estradiol (E2), ICI 182780 (ER antagonist), or RU486 (PR antagonist) treatments on ovariectomized WT mice or early pregnant WT mice were done as previously reported [22]–[24]. Briefly, the ovariectomized 6 weeks old virgin WT females (recovered for 2 weeks after surgery) were s.c. injected with 0.1 ml sesame oil (vehicle group) or 0.1 ml 20 mg/ml P4 three times on 0 h, 24 h and 48 h, respectively. In the E2-treated group, the ovariectomized mice were injected with 0.1 ml oil on 0 h and 24 h, then 0.1 ml 1 µg/ml E2 on 48 h. In the P4+E2 -treated group, the ovariectomized mice were treated the same as the P4 treated group except an additional injection of 0.1 ml 1 µg/ml E2 on 48 h. All injected mice were dissected 6 hours after the last injection. The total treatment time of P4 and E2 were 54 hours and 6 hours, respectively. Another set of ovariectomized mice were treated with 0.1 ml sesame oil (vehicle group), 0.1 ml 20 mg/ml P4, 0.1 ml 20 mg/ml P4 and 200 µg/ml RU486 (P4+RU486 group), or 0.1 ml 200 µg/ml RU486 (RU486 group), respectively. All mice were dissected 24 hours post injection and the uterine tissues were snap-frozen on dry ice. The third set of treatments was on naturally mated early pregnant mice. They were treated with 0.1 ml sesame oil (vehicle group), 0.1 ml 200 µg/ml ICI 182780 (ICI 182780 group), or 0.1 ml 200 µg/ml RU486 (RU486 group) on D2.5, and dissected on D3.5. The pregnancy status was determined as mentioned above. About 1/3 of a uterine horn from each female was snap-frozen for *in situ* hybridization. The remaining uterine horns were flushed with 1×PBS and snap-frozen for realtime PCR.

### LE isolation

D3.5 uteri from naturally mated WT and *Npl* ^(−/−)^ mice were processed for LE isolation as previously described using 0.5% dispase enzyme and gentle scraping [22]. The pregnancy status was determined by the presence of blastocyst(s). At least five pregnant mice were included in each group.

### Realtime PCR

Total RNA from whole uterine horns or LE sheets were isolated using TRIzol. cDNA was reverse-transcribed from one microgram of total RNA using Superscript III reverse transcriptase with random primers (Invitrogen, Carlsbad, CA, USA). Realtime PCR was performed in 384-well plates using Sybr-Green I intercalating dye on ABI 7900 (Applied Biosystems, Carlsbad, CA, USA) to quantify the mRNA expression levels of *Npl* and several other sialic acid metabolism related genes, including glucosamine (UDP-N-acetyl)-2-epimerase/N-acetylmannosamine kinase (*Gne*), N-acetylneuraminic acid synthase (*Nans*), N-acetylneuraminic acid phosphatase (*Nanp*), cytidine monophosphate N-acetylneuraminic acid synthetase (*Cmas*), cytidine monophospho-N-acetylneuraminic acid hydroxylase (*Cmah*), solute carrier family 35, member A1 (*Slc35a1*), sialidase 1 (*Neu1*), sialidase 3 (*Neu3*), solute carrier family 17, member 5 (*Slc17a5*), ST3 beta-galactoside alpha-2,3-sialyltransferase 1 (*St3gal1*),), ST3 beta-galactoside alpha-2,3-sialyltransferase 4 (*St3gal4*), ST8 alpha-N-acetyl-neuraminide alpha-2,8-sialyltransferase 5 (*St8sia5*). The mRNA expression levels were normalized by the expression of *Gadph* (glyceraldehyde-3-phosphate dehydrogenase). *Hprt1* (hypoxanthine phosphoribosyltransferase 1) served as the second house-keeping gene. Primer sequences (Integrated DNA Technology, San Diego, CA, USA) were shown in Table 2.

**Table 2:** Primers used for realtime PCR, making probes for *in situ* hybridization.

Primer	Sequence	Product size (bp)
*Npl*	F: GAACAAGTTGGACCAGGTG	399
	R: AGAGCACTCAGCAGTTGTTC	
*Gne*	F: GCATGATTGAGCAAGATGAC	380
	R: CGGAGAGCAGTTTGTCATAG	
*Nans*	F: TGCGCTAAGTTTCAGAAGAG	395
	R: AAGCAGAAGTTGGGATTCAG	
*Nanp*	F: TTCTTTGACCTGGACAACAC	391
	R: CCCTCTGAGTCTGTCTGTCA	
*Cmas*	F: TCCCACTGAAGAACATCAAG	380
	R: CTGAATTTCACTCCATCGAA	
*Cmah*	F: CTCAAGGAAGGGATCAATTT	399
	R: CTTGTCTCCCAACTTGAGGT	
*Slc35a1*	F: ACAAGGACAACAGCTGAAGA	382
	R: TCCACTGTACGAGTGTGACC	
*Neu1*	F: CAGATCGGCTCTGTAGACAC	380
	R: AACATCTCTGTGCCAATATCC	
*Neu3*	F: AACAGAGTGGGGTGACCTAC	384
	R: CGGTCAAGTCTTTCACTTCA	
*Slc17a5*	F: TCTGCTCGGTACAACTTAGC	394
	R: TAAGCACAACGAGAGTCACC	
*St3gal1*	F: TTCCTCACTTCCTTTGTCCT	384
	R: GTTTCCTACAACTGCACAGC	
*St3gal4*	F: CAAGACCACCATACGTCTCT	394
	R: GGTCTGCTTCTTGTTGGAG	
*St8sia5*	F: GGCTTATATCACATCGACCA	382
	R: GCATGTATTTGACTCGGAAG	
*Prap1*	F: AGGAAACAGAGAAGGTCTGG	224
	R: GTCAGACATGGGATGGTCTA	
*Abp1*	F: TACCCTAATGGTGTGATGGA	398
	R: TCAGCCATAGAGTGGATCTG	
*Dtprp*	F: GCTCAGATCCCCTTGTGAT	396
	R: GGTCATCATGGATTTCTCTG	
*Gapdh*	F: GCCGAGAATGGGAAGCTTGTCAT	230
	R: GTGGTTCACACCCATCACAAACAT	
*Hprt1*	F: GCTGACCTGCTGGATTACAT	172
	R: CAATCAAGACATTCTTTCCAGT	

F: forward primer; R: reverse primer. 5′→3′.

### *In situ* hybridization

*In situ* hybridization was performed as previously described [22], [25], [26]. Sense and antisense probes for *Npl*, proline-rich acidic protein 1 (Prap1), amiloride binding protein 1 (Abp1), decidual/trophoblast prolactin-related protein (Dtprp) were synthesized from a cDNA fragment amplified with their respective gene specific primer pairs (Table 2).

### Postnatal growth, embryo implantation, gestation period, and litter size

The postnatal body weights of WT, *Npl* ^(+/−)^, and *Npl* ^(−/−)^ pups were recorded weekly. Young virgin WT, *Npl* ^(+/−)^, and *Npl* ^(−/−)^ females (2–4 months old) were mated with WT stud males to determine the effect of *Npl*-deficiency on female reproduction. The numbers of implantation sites were recorded on D4.5. Gestation periods and litter sizes were recorded as previously described [21]. Another set of *Npl* ^(+/−)^ and *Npl* ^(−/−)^ females were mated with *Npl* ^(−/−)^ males to determine the effect of *Npl*-deficiency on embryo development. WT and *Npl* ^(−/−)^ females were mated with *Npl* ^(−/−)^ males to determine the fertility of *Npl* ^(−/−)^ males.

### Access to online data about *Npl^Gt(OST15553)Lex^*

Detail information from the original producer of *Npl* ^(−/−)^ mice about fertility, blood chemistry, cardiology, immunology and neurology, etc. of *Npl* ^(−/−)^ mice is available on the following link: http://www.informatics.jax.org/external/ko/lexicon/2492.html.

### Statistical analyses

Two-tail unequal variance Student's t-test and one-way ANOVA with Dunnett-t test were used for various comparisons. The significant level was set at p<0.05.

## Results and Discussion

### Differential expression of *Npl* in periimplantation mouse uterus

**Figure 1:**
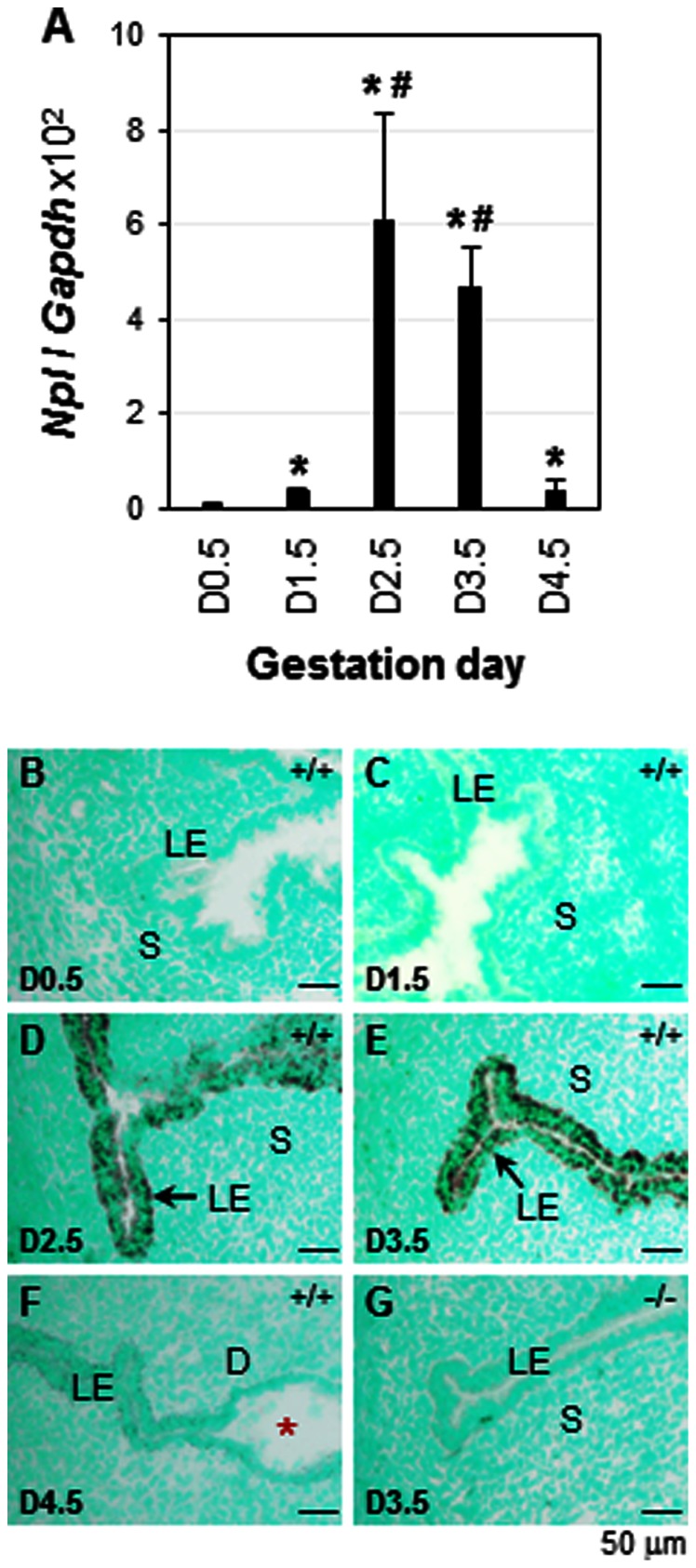
Expression and localization of *Npl* in the periimplantation mouse uterus. A. Expression of *Npl* in the periimplantation wild type (WT) uterus using realtime PCR. N = 4–6. * p<0.05, compared to gestation day 0.5 (D0.5); ^#^ p<0.05, compared to D1.5 and D4.5. *Gapdh* (glyceraldehyde 3-phosphate dehydrogenase), a house keeping gene as a loading control; error bars, standard deviation. B–G. Localization of *Npl* in the periimplantation uterus by *in situ* hybridization using *Npl* antisense probe. B. D0.5 WT (+/+) uterus. C. D1.5 WT uterus. D. D2.5 WT uterus. E. D3.5 WT uterus. F. D4.5 WT uterus. G. D3.5 *Npl* ^(−/−)^ uterus. Red star, embryo; LE, luminal epithelium; S, stroma; D, decidual zone; scale bar, 50 µm. N = 2–3.

To determine the spatiotemporal uterine expression of *Npl* during early pregnancy, the mRNA expression of *Npl* was examined in the periimplantation uterus by realtime PCR and *in situ* hybridization. Realtime PCR indicated that *Npl* was expressed at a very low level in the D0.5 uterus; it was increased 4× in the D1.5 uterus and 56× in the D2.5 uterus; its expression level was slightly lower (44×) in the D3.5 uterus compared to that in the D2.5 uterus; upon embryo implantation, *Npl* expression level in the D4.5 uterus returned to a level comparable to that of D1.5 uterus (Fig. 1A). *In situ* hybridization didn't detect any significant *Npl* signal in the D0.5 and D1.5 uterus (Figs. 1B, 1C). *Npl* was exclusively detected in the uterine luminal epithelium (LE) on D2.5 and D3.5 with comparable intensity (Figs. 1D, 1E) but undetectable in the postimplantation uterus from D4.5 to D7.5 (Fig. 1F and data not shown). One study indicated that *Npl* expression is >5× lower in the LE at the implantation site than that in the LE of inter-implantation site on D4.5 [27]. However, *in situ* hybridization on longitudinal sections of D4.5 uterus did not detect any *Npl* signal in the inter-implantation site (data not shown). It suggests that the *Npl* expression levels in the D4.5 LE from implantation site and inter-implantation site are too low to be detected by *in situ* hybridization, similar as that in the D0.5 and D1.5 uterus (Fig. 1A). The upregulation of *Npl* in the preimplantation LE was consistent with microarray (GEO number: GSE44451) and realtime PCR results (Fig. 1A).

### PR-mediated upregulation of *Npl* in preimplantation mouse uterus

**Figure 2:**
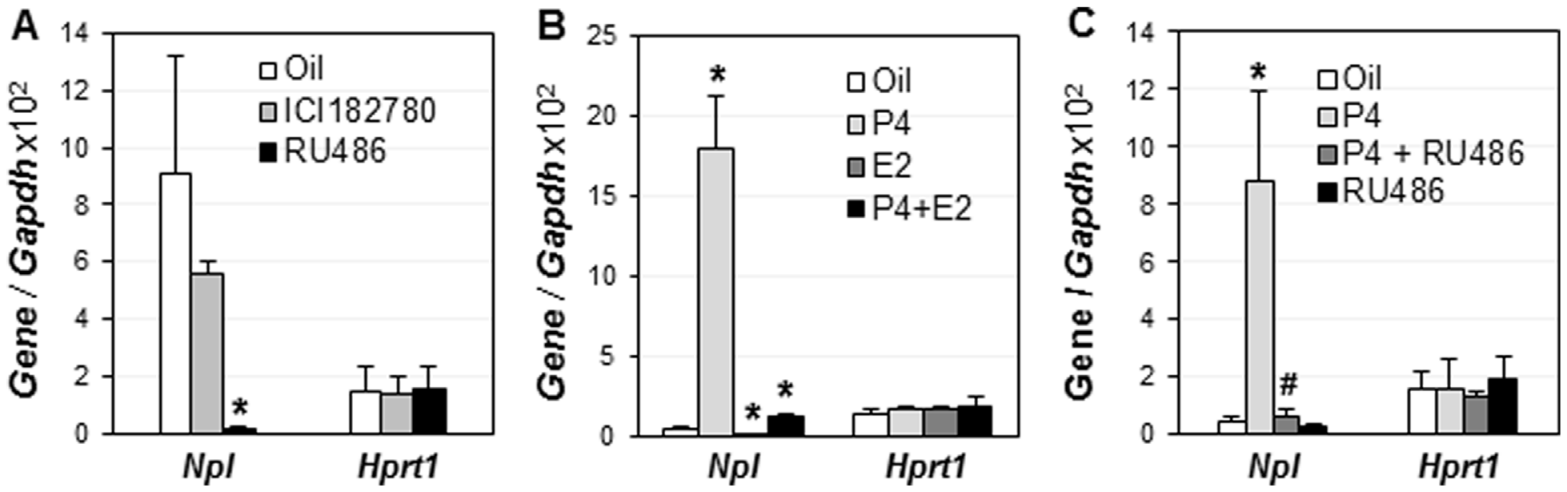
Hormone regulation of *Npl* in preimplantation and ovariectomized wild type mouse uterus using realtime PCR. A. Expression of *Npl* in the preimplantation uterus treated with estrogen receptor antagonist ICI 182780 or progesterone receptor antagonist RU486 (N = 3–4). B. Regulation of *Npl* by progesterone (P4) and 17β-estradiol (E2) in ovariectomized uterus (N = 4–6). * p<0.05, compared to oil-treated group. C. Effect of RU486 on P4 induced uterine *Npl* expression in ovariectomized mice (N = 4–5). * p<0.05, compared to oil-treated group; ^#^ p<0.05, compared to P4 treated group; *Gapdh* (glyceraldehyde 3-phosphate dehydrogenase), a house keeping gene as a loading control; *Hprt1* (hypoxanthine phosphoribosyltransferase 1), another house keeping gene; error bars, standard deviation.

Uterine gene expression is largely controlled by ovarian hormones P4 and E2, whose functions are mediated via their receptors PR and ER, respectively [28], [29]. To determine the molecular mechanism for *Npl* upregulation in the preimplantation uterus, D2.5 WT females were treated with PR antagonist RU486 or ER antagonist ICI 182780 and *Npl* expression level was analyzed in the D3.5 uterus by realtime PCR. No significant difference in *Npl* expression was observed between ICI 182780-treated and vehicle-treated groups (Fig. 2A). However, dramatically reduced *Npl* expression was observed in the RU486-treated group (53× compared to vehicle-treated control) (Fig. 2A). The expression of the house-keeping gene *Hprt1* was not changed upon ICI 182780 or RU486 treatments (Fig. 2A). These results indicated that PR mediated the upregulation of *Npl* in the preimplantation uterus.

### Hormonal regulation of *Npl* in ovariectomized mouse uterus

In ovariectomized WT mice, *Npl* was significantly upregulated (45×) by P4 treatment and downregulated (6×) by E2 treatment in the uterus (Fig. 2B). P4-induced *Npl* upregulation was greatly reduced by co-administration of E2, although the expression level of *Npl* was still 3-fold higher than that in the vehicle-treated uterus (Fig. 2B). *In situ* hybridization revealed that P4-induced upregulation of *Npl* was also detected in the LE of the ovariectomized uterus (data not shown), similar as that in the preimplantation uterus (Figs. 1D, 1E). To determine the involvement of PR in the regulation of *Npl* in the ovariectomized uterus, another set of experiment was performed. The results indicated that P4-induced upregulation of *Npl* in the ovariectomized uterus was completely abolished by co-administration of RU486, whereas RU486 alone did not seem to affect *Npl* expression (Fig. 2C). The expression of the house-keeping gene *Hprt1* was not changed upon different hormonal treatments (Fig. 2). These data demonstrated that P4-PR signaling mediated the upregulation of *Npl* in the mouse uterus.

The coordinated uterine regulation of *Npl* by both P4 and E2 could explain the temporal expression of *Npl* in the early pregnant uterus (Fig. 1A). After D1.5, P4 secretion from the newly formed corpus luteum increases [30] and correspondingly, *Npl* expression levels increase in the D2.5 and D3.5 uterus (Fig. 1). On D3.5, superimposed ovarian estrogen secretion, which makes the P4-primed uterus receptive for embryo implantation [30], [31], may contribute to the downregulation of *Npl* (Figs. 1A, 1F, 2B). However, since ER antagonist ICI 182780 does not significantly affect the expression of *Npl* in the preimplantation uterus and PR antagonist RU486 dramatically suppresses the expression of *Npl* in the preimplantation uterus (Fig. 2A), it is more likely that the downregulation of *Npl* in the postimplantation LE (Fig. 1F) is the consequence of the downregulation of PR in the postimplantation LE [26].

Since NPL catalyzes the dominant sialic acid N-acetylneuraminic acid, it is expected that its downregulation could lead to elevated sialic acid in the uterus. The downregulation of *Npl* expression upon E2 treatment in the ovariectomized mouse uterus (Fig. 2B) seems to agree with the increased uterine sialic acid concentration upon E2 treatment in the ovariectomized Indian langur monkeys [18].

LE is the first cellular layer that an implanting embryo communicates with for implantation. Considering the observations that *Npl* is the most dramatically differentially expressed gene in the periimplantation LE (Fig. 1) (Xiao S et al, submitted) and *Npl* is upregulated in the preimplantation uterus via P4-PR signaling (Fig. 2), we hypothesized that *Npl* might play a role in uterine function, especially uterine preparation for embryo implantation. This hypothesis was tested in the *Npl* ^(−/−)^ mice and appeared to be supported by the preliminary fertility data on MMRRC website, which indicated that the average litter size from *Npl* ^(−/−)^ females (mated with WT males) was 1.67±1.53 (N = 3), significantly smaller than that from WT females (mated with *Npl* ^(−/−)^ males) with an average litter size of 7.67±2.08 (N = 3, P<0.05) (http://www.informatics.jax.org/external/ko/lexicon/2492.html).

### General characterization of *Npl* ^(−/−)^ mice

Deletion of *Npl* was confirmed by genotyping and lack of *Npl* signal in both D2.5 and D3.5 *Npl* ^(−/−)^ uteri by *in situ* hybridization (Fig. 1G and data not shown). There was no significant difference in postnatal growth among WT, *Npl* ^(+/−)^, and *Npl* ^(−/−)^ mice of the same genders (data not shown). The percentages of WT, *Npl* ^(+/−)^, and *Npl* ^(−/−)^ offspring from 27 litters (224 pups at weaning) of *Npl* ^(+/−)^ and *Npl* ^(+/−)^ crosses were 24.55%, 54.91%, and 20.54%, respectively. Among them, 117 (52.23%) were females and 107 (47.77%) were males. No obvious difference in mating activities was observed between WT and *Npl* ^(−/−)^ mice in both genders.

### Normal embryo implantation and postimplantation pregnancy in *Npl* ^(−/−)^ females

**Figure 3:**
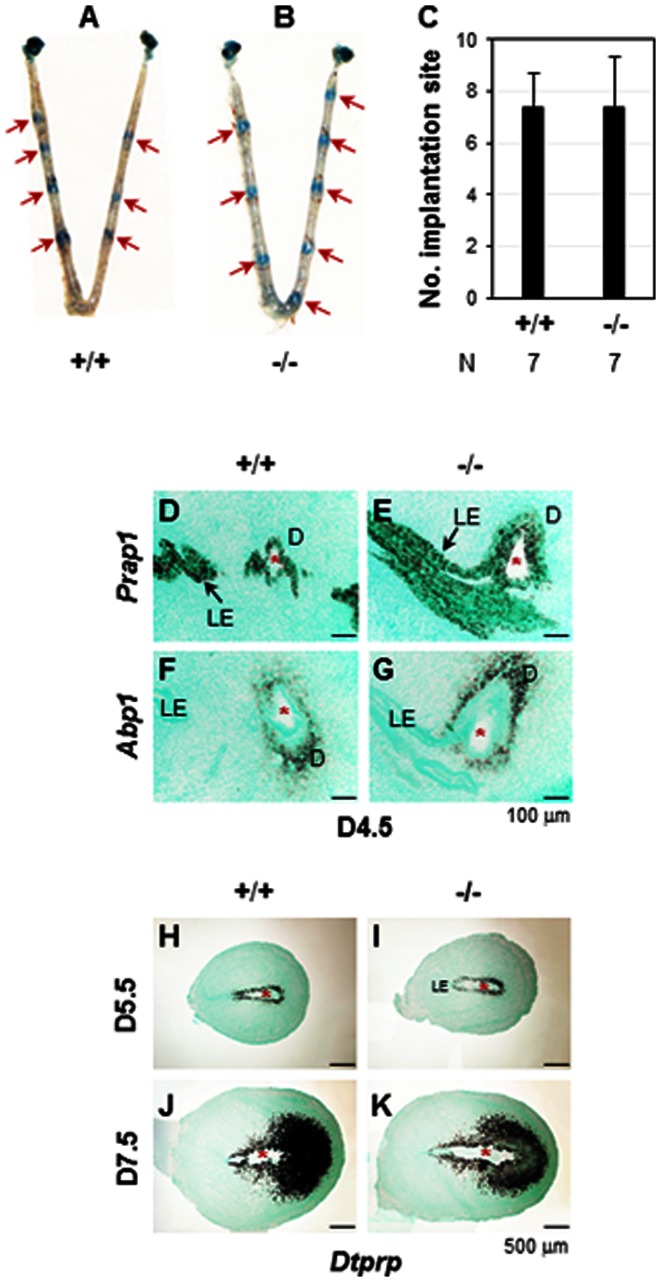
Deletion of *Npl* on embryo implantation and the expression of implantation and decidualization markers in gestation day 4.5 (D4.5), D5.5 and D7.5 uteri. *+/+*, wild type (WT); *−/−*, *Npl* ^(−/−)^. A. A representative uterus from D4.5 WT mice. B. A representative uterus from D4.5 *Npl* ^(−/−)^ mice. Red arrow, implantation site. C. the number of implantation sites on D4.5. N, the number of female mice in each group; error bars, standard deviation. D–G. Expression of implantation markers in D4.5 uterus by *in situ* hybridization using *Prap1* and *Abp1* antisense probes, respectively. D. *Prap1* in WT uterus. E. *Prap1* in *Npl* ^(−/−)^ uterus. F. *Abp1* in WT uterus. G. *Abp1* in *Npl* ^(−/−)^ uterus. H–K. Expression of decidualization marker, *Dtprp*, in D5.5 and D7.5 uterus by *in situ* hybridization using *Dtprp* antisense probes. H. *Dtprp* in D5.5 WT uterus. I. *Dtprp* in D5.5 *Npl* ^(−/−)^ uterus. J. *Dtprp* in D7.5 WT uterus. K. *Dtprp* in D7.5 *Npl* ^(−/−)^ uterus. Red star, embryo; LE, luminal epithelium; D, decidual zone; scale bar, 100 µm (D–G) and 500 µm (H–K). N = 2–3. No signals were detected using *Prap1*, *Abp1*, or *Dtprp* sense probes (data not shown).

**Figure 4:**
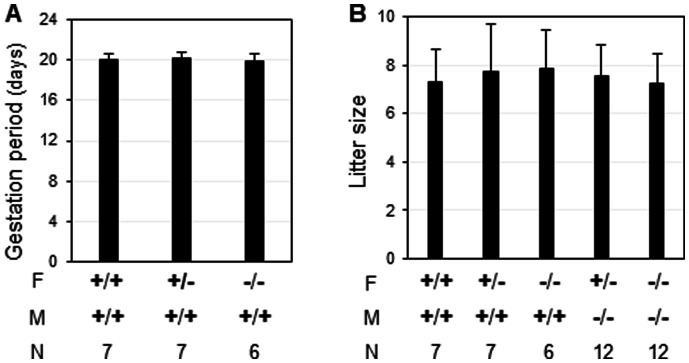
Deletion of *Npl* on gestation periods (days) from females with different genotypes crossed with wild type males (A), and litter sizes (B). *+/+*, wild type; *+/−*, *Npl* ^(+/−)^; *−/−*, *Npl* ^(−/−)^; F, female; M, male; N, the number of female mice in each group; error bars, standard deviation.

Embryo implantation initiates around D4.0 in mice [26]. On D4.5, all the pregnant *Npl* ^(−/−)^ females had implantation sites detected by blue dye injection as seen in the WT females (Figs. 3A, 3B). The intensity and spacing of the blue bands, which indicated the implantation sites, and the average numbers of implantation sites between WT and *Npl* ^(−/−)^ females were comparable (Figs. 3A–3C). These data indicated no obvious defect in embryo implantation, which was confirmed by the comparable uterine expression of *Prap1* and *Abp1* (Figs. 4D–4G), a uterine LE marker [25] and a decidualization marker [32] upon embryo implantation, respectively. These results also indicated that all the preimplantation events, including oogenesis, ovulation, fertilization, embryo transport, and preimplantation embryo development, were not impaired. Postimplantation decidualization was also well developed in the D5.5 and D7.5 *Npl* ^(−/−)^ uteri, demonstrated by the comparable expression of a decidualization marker *Dtprp* [33] in D5.5 and D7.5 WT and *Npl* ^(−/−)^ uterus (Figs. 3H–3K). In fact, no obvious defect was detected in the *Npl* ^(−/−)^ females during the entire pregnancy, revealed by the comparable gestation periods, litter sizes, survival rates, and postnatal growth of offspring from females with different genotypes when they were mated with WT males (Figs. 4A, 4B, and data not shown). These results proved our hypothesis wrong and didn't support the preliminary fertility data reported in the MMRRC website (http://www.informatics.jax.org/external/ko/lexicon/2492.html).

### Non-essential role of *Npl* in male fertility and embryo development

The WT and *Npl* ^(−/−)^ males had comparable testis weight, sperm counts from cauda epididymis, and litter sizes when they were mated with WT females (data not shown), indicating normal fertility of *Npl* ^(−/−)^ males.

When *Npl* ^(+/−)^ or *Npl* ^(−/−)^ females were mated with *Npl* ^(−/−)^ males, they produced comparable litter sizes to that from WTxWT crosses (Fig. 4B). These data demonstrated that deletion of *Npl* did not have an obviously adverse effect on embryo development.

### No compensatory mRNA expression of other genes involved sialic acid metabolism in D3.5 *Npl* ^(−/−)^ uterus

The following genes are known to play roles in sialic acid metabolism: *Gne*, *Nans*, and *Nanp*, which are important for the sialic acid synthesis from the UDP-N-acetylglucosamine (UDP-GlcNAc) to Neu5Ac in cytosol; *Cmas* and *Cmah*, which catalyze Neu5AC to CMP-Neu5Ac and then Neu5Gc; *Slc35a1*, which transports CMP-Neu5Ac and CMP-Neu5Gc to Golgi compartment for further glycosylation; *St3gal1* and *St3gal4*, which are sialytransferases controlling the glycosylation of sialic acid with carbohydrates, glycoproteins, and glycolipids; *Neu1* and *Neu3*, which are neuraminidases responsible for the removal of sialic acid residues from glycoconjugates in different intracellular compartments, such as lysosome, plasma membrane, and mitochondria; *Slc17a5*, which transports free sialic acid back to the cytosol; and *Npl*, which degrades the free sialic acid to N-acetylmannosamine and pyruvate in the cytosol [7], [34].

**Figure 5:**
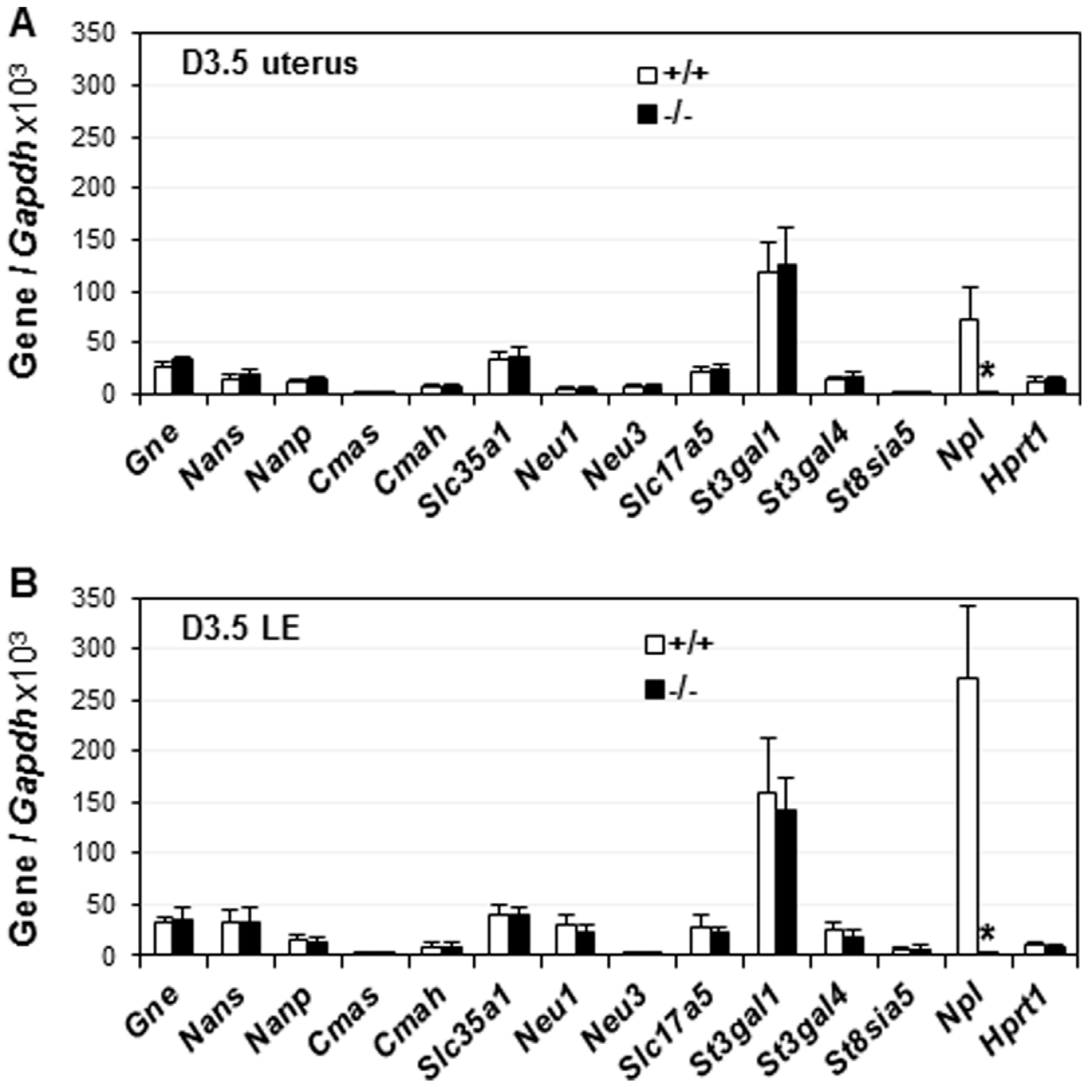
Deletion of *Npl* on the expression of sialic acid metabolism related genes in D3.5 uterus (A) and isolated LE (B), respectively. *+/+*, wild-type; *−/−*, *Npl* ^(−/−)^; X-axis indicated the names of the examined genes. Y-axis showed the normalized mRNA expression levels by *Gapdh* (glyceraldehyde 3-phosphate dehydrogenase) x10^3^. *Hprt1* (hypoxanthine phosphoribosyltransferase 1) served as the second house-keeping gene. N = 5–6. Error bars, standard deviation.

Since *Npl* expression peaks in the preimplantation uterine LE (Fig. 1), the mRNA expression levels of the above mentioned genes involved in sialic acid metabolism were examined in the preimplantation D3.5 WT and *Npl* ^(−/−)^ uteri by realtime RT-PCR. The results showed comparable mRNA expression levels of all these genes between WT and *Npl* ^(−/−)^ uteri (Fig. 5A). Since *Npl* is an LE-specific gene (Fig. 1) and LE comprises <10% of the uterine cells [19], [20], [35], any compensatory mRNA changes of these genes in the LE could potentially be covered in the whole uterine gene expression analysis. Therefore, LE cells from D3.5 WT and *Npl* ^(−/−)^ uteri were isolated for determining the mRNA expression of these genes. Realtime RT-PCR still failed to detect any significant difference of these genes between D3.5 WT and *Npl* ^(−/−)^ LE (Fig. 5B). *Npl* was included in both uterine and LE analyses (Figs. 5A, 5B) to indicate the deletion of *Npl* in the *Npl* ^(−/−)^ uterus. Based on the relative expression levels compared to the house-keeping gene *Gapdh*, *Npl* was enriched in the LE (Fig. 5), consistent with the *in situ* data (Fig. 1E). These results indicated no compensatory mRNA expression of these genes involved in sialic acid metabolism in the D3.5 *Npl* ^(−/−)^ whole uterus (Fig. 5A) and LE (Fig. 5B).

It has been reported that disruption of sialic acid metabolism and transport could have adverse effects. In mice, inactivation of *Gne*, which is important for sialic acid synthesis in the cytosol, leads to early postnatal lethality [36]. In humans, mutations of *Slc17a5*, which transports the free sialic acid from lysosome to cytosol for further degradation, could lead to the sialic acid storage disease (SASD) caused by sialic acid accumulation in the lysosome, and developmental delays and growth retardation [37]. In bacterial species, mutations of *Npl*, which degrades sialic acid in the cytosol, could lead to toxic overexpression of sialic acid [38]. These results indicate that balanced sialic acid metabolism and compartmentalization are critical for normal physiological functions.

Normal embryo implantation in the *Npl* ^(−/−)^ females (Fig. 3) indicates that NPL is not essential for embryo implantation. However, being the most downregulated gene in the postimplantation LE implies that it might have redundant, although nonessential, roles in uterine preparation for embryo implantation. Our unpublished microarray data demonstrated that *Npl* expression was significantly decreased in the pregnant mouse uterus compared to that in the pseudopregnant mouse uterus at 22:00 h on D3.5 (data not shown), right before embryo attachment to the LE for implantation indicated by blue dye reaction [26], suggesting that downregulation of *Npl* might contribute to the initiation of embryo attachment. Since NPL degrades sialic acid that is involved in cell adhesion [39], it is possible that downregulation of *Npl* could potentially facilitate embryo attachment to the LE for embryo implantation. On the other hand, sialic acid can block the access of antigenic molecules to the cell surface [40] while NPL from C. *perfringens* can dramatically increase (25×) the capacity of B cell antigen presentation [41]. It is possible that NPL might be involved in modulating the uterine immune response during early stages of embryo implantation [42].

In summary, this study demonstrates PR-mediated spatiotemporal expression of *Npl* in the periimplantation mouse uterus and the nonessential role of *Npl* in uterine function and embryo development.
